# Modifying Effects of Vitamin E on Chlorpyrifos Toxicity in Atlantic Salmon

**DOI:** 10.1371/journal.pone.0119250

**Published:** 2015-03-16

**Authors:** Pål A. Olsvik, Marc H. G. Berntssen, Liv Søfteland

**Affiliations:** National Institute of Nutrition and Seafood Research, Bergen, Norway; Huazhong university of Science and Technology, CHINA

## Abstract

The aim of this study was to elucidate how vitamin E (alpha tocopherol) may ameliorate the toxicity of the pesticide chlorpyrifos in Atlantic salmon. Freshly isolated hepatocytes were exposed to vitamin E, chlorpyrifos or a combination of vitamin E and chlorpyrifos (all 100 μM). Transcriptomics (RNA-seq) and metabolomics were used to screen for effects of vitamin E and chlorpyrifos. By introducing vitamin E, the number of upregulated transcripts induced by chlorpyrifos exposure was reduced from 941 to 626, while the number of downregulated transcripts was reduced from 901 to 742 compared to the control. Adding only vitamin E had no effect on the transcriptome. Jak-STAT signaling was the most significantly affected pathway by chlorpyrifos treatment according to the transcriptomics data. The metabolomics data showed that accumulation of multiple long chain fatty acids and dipeptides and amino acids in chlorpyrifos treated cells was partially alleviated by vitamin E treatment. Significant interaction effects between chlorpyrifos and vitamin E were seen for 15 metabolites, including 12 dipeptides. The antioxidant had relatively modest effects on chlorpyrifos-induced oxidative stress. By combining the two data sets, the study suggests that vitamin E supplementation prevents uptake and accumulation of fatty acids, and counteracts inhibited carbohydrate metabolism. Overall, this study shows that vitamin E only to a moderate degree modifies chlorpyrifos toxicity in Atlantic salmon liver cells.

## Introduction

Several nutrients have modifying effects on the toxicity of contaminants. Interactions between nutrients and contaminants can enhance the protection against negative effects of unwanted compounds. For example, vitamin E (tocopherols), flavonoids, and fatty acids amend the toxicity of polycyclic aromatic hydrocarbons (PAHs) and pesticides [1,2,3]. The mechanisms underlying such interactions are however often not very well characterized.

Interactions between nutrients and contaminants are of particular interest in fish, and especially in farmed fish such as Atlantic salmon, with the recent replacement of fish-based feed ingredients with plant-based feed ingredients. Due to overfishing and reduced global stocks of fish used to produce commercial feeds [4], the farming industry today uses salmon diets >60% of the fish oil replaced with plant oil, and with decreasing fishmeal inclusion levels [5]. Dietary fatty acid composition can affect tissue fatty acids in Atlantic salmon in a tissue-dependent pattern [6], especially in liver and white muscle [7], and compromise the immune system of the fish [8]. In addition, while fish-based oils are rich in alpha-tocopherol, plant-based feeds may contain more gamma-tocopherol. These forms of vitamin E, which have antioxidant and anti-inflammatory properties, may have different abilities to protect the fish against the effects of contaminants.

With increasing inclusion of plant-based ingredients, feeds for Atlantic salmon may become contaminated with agricultural pesticides. Recently, wide-scale screening of fish feeds for contaminants has identified, among others, the insecticides chlorpyrifos-methyl and pirmiphos-methyl as possible threats for farmed salmon [9]. New plant-based feeds thus not only alter the dietary balance of essential nutrients and change the nutritional composition of the fish, but also introduce contaminants not normally associated with salmon farming. The organophosphate pesticide chlorpyrifos is a broad-spectrum insecticide used to kill a wide variety of insects [10]. It remains one of the most widely used agricultural organophosphate insecticides, and is currently in use in more than 100 countries worldwide [11,12,13]. Chlorpyrifos is highly toxic to aquatic organisms including fish. It bioaccumulates in fish and has a 96-hour LC50 toxicity value in rainbow trout (*Oncorhynchus mykiss*) between 7.1 and 51 μg/L, depending on water temperature [14]. Chlorpyrifos has at least three main modes of action in mammals. It inhibits the enzyme acetylcholinesterase (AChE), causes oxidative stress and endocrine disruption [15]. In both mammals and fish, AChE inhibition is the main effect of chlorpyrifos exposure [16]. The main detoxification system is via the cytochrome P450 enzyme system [17]. Chlorpyrifos is completely metabolized to chlorpyrifos oxon and then to 3,5,6-trichloro-2-pyridinol (TCP) in the mammalian liver by cytochrome P450 system [18].

The aim of this study was to evaluate whether vitamin E (alpha tocopherol) amends the toxicity of chlorpyrifos in Atlantic salmon. Vitamin E has been reported to be partially protective against chlorpyrifos in animal models [19,20,21]. Freshly isolated primary hepatocytes were used as a model, and cells were either kept as control or treated with 100 μM alpha tocopherol, 100 μM chlorpyrifos or a combination of vitamin E and chlorpyrifos for 48 hours. Previous experiments have shown the chosen chlorpyrifos concentration to be non-cytotoxic to Atlantic salmon hepatocytes, but still high enough to induce marked transcriptional responses [22]. Similar experiments have been conducted with vitamin E, providing the rationale for using the selected concentration of alpha tocopherol in the current study (unpublished data). Direct sequencing (RNA-seq) and metabolite profiling were used to screen for interactive effects between vitamin E and chlorpyrifos. To evaluate whether transcriptional profiling can be used to predict the metabolite outcome in the cells, we compared the significantly affected KEGG pathways, as identified with transcriptional profiling, with the predicted cellular responses based on affected metabolites.

## Materials and Methods

### Fish sampling and cell harvesting

Fish sacrifice was conducted by the authors and approved by the Norwegian Animal Research Authority (NARA) via NIFES' Animal Care and Use Committee. Juvenile Atlantic salmon (*Salmo salar*) were obtained and kept at the animal holding facility at Industrilaboratoriet (ILAB), Bergen, Norway. The fish were fed once a day a special feed produced without synthetic antioxidants and with low levels of contaminants, delivered by EWOS, Norway (Spirit 400-50A HH, 6.0 mm). All glassware, instruments and solutions were autoclaved prior to liver perfusion. Hepatocytes were isolated from 6 male Atlantic salmon (mean±SEM: 555±20 g) with a two-step perfusion method earlier described by Søfteland et al. [23]. The fish were sacrificed by terminal anaesthetization with tricaine methanesulfonate (MS-222) (200 mg/l). Harvesting of cells was conducted in agreement with and approved by national legislation. The final cell pellet was resuspended in L-15 medium containing 10% Fish serum (FS) from salmon (Nordic BioSite, Oslo, Norway), 1% glutamax (Invitrogen, Norway) and 1% penicillin-streptomycin-amphotericin (10000 units/ml potassium penicillin 10000 mcg/ml steptomycin sulfate and 25 μg/ml amphotericin B.) (Lonzo, Medprobe, Oslo, Norway). The Trypan Blue exclusion method was performed in accordance with the manufacturer’s protocol (Lonzo, Medprobe, Oslo, Norway) and was used to determine cells viability. The cell suspensions were plated on 2 μg/cm^2^ laminin (Sigma-Aldrich, Oslo, Norway) coated culture plates (TPP, Trasadingen, Switzerland) and the hepatocytes were kept at 10°C in a sterile incubator without additional O_2_/CO_2_ (Sanyo, CFC FREE, Etten Leur, Netherland). The following cell densities were used; 7.2×10^6^ cells per well in 6-well plates (in 3 ml complete L-15 medium for transcriptional and metabolite profiling) and 0.2×10^6^ cells per well in xCELLigence 96-well plates (in 0.2 ml complete L-15 medium for cytotoxicity screening).

### Exposure experiments

The cells were cultured for 36–40 h prior to chemical exposure with exchange of medium (containing 10% FS) after 18–20 hours. For the exposure experiments, cells were treated with alpha tocopherol (100 μM), chlorpyrifos (100 μM) or a combination of alpha tocopherol (100 μM) and chlorpyrifos (100 μM) and harvested after 48 hours exposure. Table 1 shows an overview of the number of samples collected for the two experiments. Chlorpyrifos was dissolved in DMSO. An equal amount of DMSO was used in all four experimental groups. Alpha tocopherol and chlorpyrifos were obtained from Sigma (Sigma-Aldrich, Oslo, Norway). The cells were exposed in triplicate wells using 6-well culture plates for the transcriptional and metabolite profiling, and in single 96-wells culture for the xCELLigence cytotoxicity screening. The exposure medium contained 1% FS. The exposure medium was exchanged with new medium after 18–20 hours and the chemical exposure was sustained for another 24 hours.

**Table 1:** Number of samples used for the various analytical methods.

Group	RNA-seq (cells)	Metabolomics (cells)	Metabolomics exposure medium	Description
Control	6	6	1	Control (w/DMSO)
Alpha tocopherol (100 μM)	6	6	1	Vitamin E (Vit E) exposed (w/DMSO)
Chlorpyrifos (100 μM)	6	6	1	Chlorpyrifos (CPF) exposed (w/DMSO)
Chlorpyrifos (100 μM) + Alpha tocopherol (100 μM)	6	6	1	Chlorpyrifos/vitamin E exposed (w/DMSO)

### Cytotoxicity screening

Impedance-based real time detection of cellular viability was conducted using the xCELLigence system (Real-Time Cell Analyzer RTCA-SP, ACEA Biosciences, San Diego, USA), described in detail by Abassi et al. [24]. Recording of cell index (CI) values and normalization was performed using the RTCA Software version 1.2.1. Primary hepatocyte cells were evenly distributed to 96-well E plates. Each well contained about 0.2 million cells. Coating and cell density optimization was ensured by preliminary experiments. The cells were allowed to attach to the 96-well E plates at room temperature (30 min) before being inserted in the cell incubator for continuous impedance recording. The real time cell monitoring was conducted at 10°C in an incubator without additional O_2_/CO_2_ (Sanyo, CFC FREE, Etten Leur, Netherland), using the RTCA single plate xCELLigence platform. The data was collected with intervals of 2 min after compound exposure for 12 hours, and then every 15 min for 60 hours. For calculation of cell viability after 48 hours of exposure, the impedance signal was analyzed by normalizing data of each singe well to a reference time point set about one hour before the final exchange of exposure medium, or after about 38 hours of exposure: CI_(normalized)_ = CI_time x_/CI_norm time_ (termed “normalized cell index” or NCI). The NCI values were calculated from cells obtained from 6 different male fish (n = 6). Determination of cytotoxic effect was done according to the International standardized test for in vitro cytotoxicity ISO 10993-5:2009 [25].

### RNA isolation

Hepatocyte cells from Atlantic salmon were treated with lysis buffer before homogenization with the Precellys 24 homogenizer by using ceramic beads CK28 (Bertin Technologies, Montigny-le-Bretonneux, France). The RNeasy Plus mini kit (Qiagen, Crawley, UK) was used to extract total RNA according to the manufacturer’s protocol. The RNA was eluted in 30 μl RNase-free MilliQ H_2_O and stored at −80°C before further processing. RNA quality and integrity were assessed with the NanoDrop ND-1000 UV-Vis Spectrophotometer (NanoDrop Technologies, Wilmington, DE, USA) and the Agilent 2100 Bioanalyzer (Agilent Technologies, Palo Alto, CA, USA). The 260/280 and 260/230 nm ratios were 2.15 ± 0.00 and 2.20 ± 0.05 in cells, respectively (n = 24, mean ± SEM). The RNA 6000 Nano LabChip kit (Agilent Technologies, Palo Alto, CA, USA) was used to evaluate the RNA integrity of the samples. The RNA integrity number (RIN) was 9.5 ± 0.1 (n = 24) in liver (mean ± SEM).

### Direct RNA sequencing (RNA-seq)

Direct RNA sequencing (RNA-seq) was used to screen for transcripts possibly affected by vitamin E (alpha tocopherol) and chlorpyrifos exposure in 24 Atlantic salmon hepatocyte cell cultures (about 7 mill. cells per culture). Poly (A) mRNA was isolated using magnetic beads with oligo (dT) from total RNA obtained from 24 cell cultures. Fragmentation buffer was added to shred mRNA to short reads. Using these short fragments (about 200 bp) as templates, random hexamer primers were applied to synthesize first-strand cDNA. Second-strand cDNA was synthesized using buffer, dNTPs, RNaseH, and DNA polymerase I. QiaQuick PCR extraction kit (Qiagen) was used to purify short double-stranded cDNA fragments. These fragments were then resolved with EB buffer for end reparation, added poly (A), and then ligated to the sequencing adapters. After agarose gel electrophoresis, the suitable fragments were selected for PCR amplification as templates. Finally, the libraries were sequenced using Illumina HiSeq 2000 (San Diego, CA, USA).

An Atlantic salmon transcriptome assembled from liver tissue was used as a reference for alignment of the RNA-seq data (24 samples, 12 millions reads per sample). Unigenes were annotated with Blastx alignment between unigenes and the databases of NR, NT, SwissProt, KEGG, COG and GO. The DESeq software package was used to screen for differentially expressed genes (DEGs). The DESeq package is based on the negative binomial distribution, and provides a method to test for differential expression by use of a shrinkage estimator for the variance. We used P-adjustment ≤ 0.5 and the absolute value of log_2_ ratio ≥ 1 as the threshold to judge the significance of gene expression difference. All RNA-seq work was performed by staff at the Beijing Genome Institute (BGI, Hong Kong).

### Metabolite profiling

Global biochemical profiles were determined in 24 Atlantic salmon hepatocyte cell cultures. Each sample contained about 7 million cells. Samples were extracted and prepared for analysis using Metabolon’s standard solvent extraction method. The extracted samples were split into equal parts for analysis on GC/MS and LC/MS/MS platforms. The LC/MS portion of the platform was based on a Waters ACQUITY UPLC and a Thermo-Finnigan LTQ mass spectrometer, which consisted of an electrospray ionization (ESI) source and linear ion-trap (LIT) mass analyzer. The sample extract was split into two aliquots, dried, and then reconstituted in acidic or basic LC-compatible solvents, each of which contained 11 or more injection standards at fixed concentrations. One aliquot was analyzed using acidic positive ion optimized conditions and the other using basic negative ion optimized conditions in two independent injections using separate dedicated columns. Extracts reconstituted in acidic conditions were gradient eluted using water and methanol both containing 0.1% Formic acid, while the basic extracts, which also used water/methanol, contained 6.5mM Ammonium Bicarbonate. The MS analysis alternated between MS and data-dependent MS^2^ scans using dynamic exclusion. The samples destined for GC/MS analysis were re-dried under vacuum desiccation for a minimum of 24 hours prior to being derivatized under dried nitrogen using bistrimethyl-silyl-triflouroacetamide (BSTFA). The GC column was 5% phenyl and the temperature ramp was from 40° to 300°C in a 16 minute period. Samples were analyzed on a Thermo-Finnigan Trace DSQ fast-scanning single-quadrupole mass spectrometer using electron impact ionization. Accurate Mass Determination and MS/MS fragmentation (LC/MS/MS) was based on Waters ACQUITY UPLC and a Thermo-Finnigan LTQ-FT mass spectrometer, which had a linear ion-trap (LIT) front end and a Fourier transform ion cyclotron resonance (FT-ICR) mass spectrometer backend. For ions with counts greater than 2 million, an accurate mass measurement could be performed. Accurate mass measurements could be made on the parent ion as well as fragments. The typical mass error was less than 5 ppm. Ions with less than two million counts require a greater amount of effort to characterize. Fragmentation spectra (MS/MS) were typically generated in data dependent manner, but if necessary, targeted MS/MS could be employed, such as in the case of lower level signals. Instrument variability was 4% for internal standards and total process variability for endogenous metabolites was 12%. The bioinformatics system consisted of four major components, the Laboratory Information Management System (LIMS), the data extraction and peak-identification software, data processing tools for QC and compound identification, and a collection of information interpretation and visualization tools for use by data analysts. Identification of known chemical entities was based on comparison to metabolomic library entries of purified standards. The metabolomics work was done by employees at Metabolon, USA.

### Statistics

Gene expression levels were calculated by using the Reads per kb per million reads (RPKM) method [26]. Screening of differentially expressed genes (DEGs) was done with pairwise comparison using the DESeq software [27]. GO annotation was obtained from blastx searches against the NR database of NCBI by using BLAST2GO with default parameters (blastx E10^−5^). Pathways enrichment analysis was done by annotation with the KEGG database (blastx E10^−5^). Following normalization to total protein (Bradford assay) and log transformation, ANOVA contrasts were used to identify biochemicals that differed significantly between experimental groups. Analysis by two-way ANOVA identified biochemicals exhibiting significant interactions and main effects for experimental parameters of dietary nutrient or contaminant. Welch’s two-sample *t*-tests were used to identify biochemicals/metabolites that differed significantly between experimental groups (P<0.05). Correction for multiple testing was done with false discovery rate (FDR) using q-values (P-adj) [28]. Statistical analyses of the log-transformed data were performed with the program “R” [29]. The functional pathway analyses were generated through the use of IPA [30].

## Results

### Exposure experiment and cytotoxicity

The different cell suspensions used in this study had cell viability between 89–96% as determined by the Trypan Blue exclusion method. After 48 hours of exposure, no significant effect of chlorpyrifos treatment was observed on cytotoxicity measured with the xCELLigence system. Neither did vitamin E supplementation, alone nor in combination with chlorpyrifos, have any effect on cytotoxicity (data not shown).

### Transcriptomics results

On average, 12,085,418±62,567 single-end Illumina reads were sequenced from the cell culture samples (n = 24, mean±SEM). The reads were mapped to a liver Atlantic salmon transcriptome assembled from 65,863,720 paired-end Illumina reads. Total mapped reads were 10,163,717±62,277 (n = 24, mean±SEM) or about 84%. The reference transcriptome was made from a pool of total RNA obtained from liver of four juvenile Atlantic salmon. Assembly of the transcriptome created 156,417 contigs with an average length of 266 nucleotides (nts) and 65,519 unigenes with an average length of 571 nts, as well as 45,855 singletons. The number of unigenes annotated in various databases were: NR: 32,699, NT: 41,578, Swiss-Prot: 29,033, KEGG: 23,356, COG: 9,486, and GO: 22,371.

Table 2 shows an overview of the number of differentially expressed genes (DEGs) according to the transcriptional profiling. The DESeq method was used with a log2 ratio ≥1 (>2-fold change) and FDR-adjusted P-values (P-adj) <0.50 and <0.10. In the pairwise comparison (A versus B), the former one (A) is considered as the control, and the latter one (B) is considered as the treatment. Exposure to 100 μM chlorpyrifos significantly upregulated 942 transcripts and downregulated 901 transcripts. Treatment with 100 μM alpha tocopherol alone did not significantly affect any transcripts in the cells. Compared to the control, by adding alpha tocopherol to chlorpyrifos the number of significantly upregulated transcripts was lowered from 942 to 626 and the number of significantly downregulated transcripts was lowered from 901 to 742. A direct comparison between the chlorpyrifos and vitamin E treatment groups showed 636 upregulated and 834 downregulated transcripts. The comparison between the vitamin E and vitamin E plus chlorpyrifos treatment groups produced 495 upregulated and 399 downregulated transcripts. S1 Dataset shows the gene lists from these pairwise comparisons. Using a stricter P-adj cut-off of <0.1, the corresponding numbers of significantly regulated genes from the pairwise comparisons were Control vs. CPF: 351 genes upregulated/182 genes downregulated, Control vs. CPF+VitE: 228 upregulated/140 downregulated, and CPF vs. VitE: 107 upregulated/306 downregulated. Since the primary goal of the transcriptional profiling was to identify biological processes, pathways and ontology categories, and not to identify specific biomarkers, gene lists created with the less strict P-adj cut-off was selected for downstream IPA analyses. With this approach, about 45% of the DEGs listed in S1 Dataset and used for downstream analyses were identified with annotation (blastx search against the NR database, cut-off E10^–5^).

**Table 2:** Number of affected transcripts and metabolites using two different statistical stringencies.

Statistical comparisons								
*Significantly altered transcripts/metabolites*	Total transcripts Log2≥1 P-adj<0.50	Transcripts (⇵)	Total transcripts Log2≥1 P-adj<0.10	Transcripts (⇵)	Total metabolites P≤0.05	Metabolites (⇵)	Total metabolites P<0.05<P-adj<0.10	Metabolites (⇵)
***Vit E***	0	0|0	0	0|0	1	0|1	3	0|3
***Control***								
***CPF***	1843	942|901	533	351|182	155	130|25	25	21|4
***Control***								
***Vit E + CPF***	1368	626|742	368	228|140	117	81|36	36	18|18
***Control***								
***CPF***	1470	636|834	413	107|306	154	135|19	30	23|7
***Vit E***								
***Vit E + CPF***	894	495|399	221	164|57	87	58|29	51	37|14
***Vit E***								
***Vit E + CPF***	0	0|0	0	0|0	32	0|32	28	1|27
***CPF***								

CPF = Chlorpyrifos. Vit E = Vitamin E.

**Fig 1:**
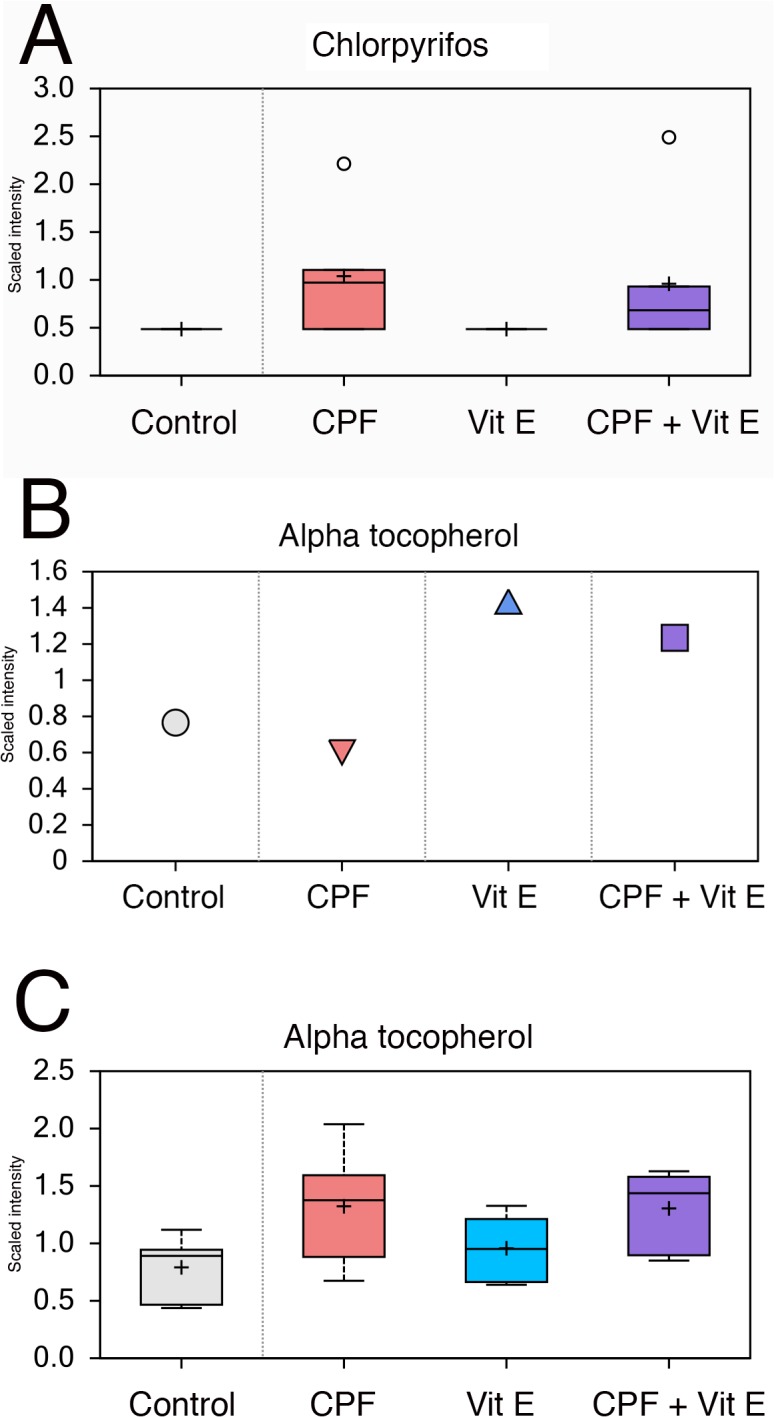
Predicted cellular outcome of chlorpyrifos exposure (100 μM) in Atlantic salmon hepatocytes based on affected transcripts. Effects on A) Jak-STAT signaling (KEGG pathway ID ko04630) and B) Protein processing in endoplasmic reticulum (ko04141). Colored enzymes: green for downregulated genes, red for upregulated genes.

KEGG pathway enrichment analysis identified JAK-STAT signaling (pathway ID ko04630) as the most significantly affected system by chlorpyrifos exposure in Atlantic salmon hepatocytes (Fig. 1A), followed by Protein processing in endoplasmic reticulum (ko04141) (Fig. 1B), Pathways in cancer (ko05200) and Osteoclast differentiation (ko04380). Statistically significant KEGG pathways (P<0.05) from gene set enrichment analysis are shown in S2 Dataset. Pathways linked to typical human diseases and of little relevance for fish were omitted from the lists. Addition of vitamin E to chlorpyrifos appears to have a limited impact on these molecular mechanisms and others according to the KEGG pathway enrichment analysis. Antigen processing and presentation (ko04612) became more significantly affected by the inclusion of vitamin E in the exposure media, but most of the pathways appear on both lists although with slightly different significance levels. Vitamin E supplementation amended the following significantly changed pathways, Carbohydrate digestion and absorption (ko04973), Aldosterone-regulated sodium reabsorption (ko04960), Glycosaminoglycan biosynthesis—chondroitin sulfate (ko00532) (significantly affected by chlorpyrifos alone, but not with co-treatment with vitamin E), and Focal adhesion (ko04510), MAPK signaling pathway (ko04010), Ubiquitin mediated proteolysis (ko04120), Other types of O-glycan biosynthesis (ko00514), D-Glutamine and D-glutamate metabolism (ko00471), Regulation of actin cytoskeleton (ko04810) and ErbB signaling pathway (ko04012) (significantly affected with vitamin E supplementation, but not by chlorpyrifos alone).

### Metabolomics results

**Fig 2:**
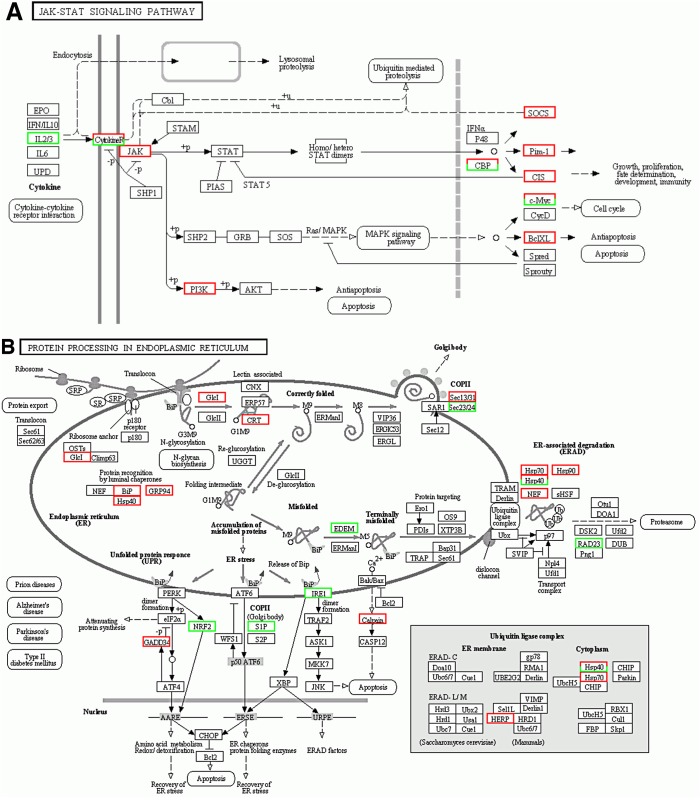
Scaled intensity levels of A) chlorpyrifos and B) alpha tocopherol (vitamin E) in Atlantic salmon hepatocytes exposed to chlorpyrifos (100 μM), alpha tocopherol (100 μM) or combined (100 μM of both). The figures show boxplots with median, upper and lower quartiles, max and min distribution, mean value (+) and extreme data points (ο).

A total of 28 samples were analyzed for metabolites, 24 cell culture samples and four exposure medium samples. Cell extracts (n = 6 per group) were normalized to Bradford protein values prior to analysis. From analysis of the dataset a total of 329 named metabolites were detected from the metabolite profiling. Vitamin E and chlorpyrifos interacted significantly on 15 metabolites (Table 3), while 9 metabolites were significantly affected by vitamin E alone and 196 by chlorpyrifos alone (2-way ANOVA). Table 2 shows an overview of the number of differentially regulated metabolites between the four treatment groups determined by Welch's two-sample t-test and using two levels of statistical stringency (P<0.05 or P<0.05 and P-adj<0.1). With the former stringency, 130 metabolites were upregulated and 25 downregulated by chlorpyrifos exposure compared to the control. Supplementation of vitamin E reduced the number of significantly upregulated metabolites 130 to 81, while at the same time increased the number of downregulated metabolites from 25 to 36. Compared to the control, vitamin E supplementation alone only significantly downregulated 1 metabolite (2-stearoylglycerophosphoinositol). Principal component analysis (PCA) revealed a separation between the control and chlorpyrifos-treated cells regardless of vitamin E treatment. Heterogeneity was observed within sample groups suggesting differences in basal metabolism between these primary-derived cells obtained from six different male fish. Chlorpyrifos-treated cells also sorted separately by hierarchical clustering compared to the control. This trend was independent of vitamin E suggesting this antioxidant may have a limited impact on the metabolic profile of these hepatocytes. Chlorpyrifos was detected in cell extracts (Fig. 2A), but not exposure media suggesting this metabolite was below the limit of detection in these samples. Alpha tocopherol was detected in exposure media (Fig. 2B) and in the cells (Fig. 2C). Pathway-specific heat maps of affected metabolites, with fold changes and significance levels, are shown in S3 Dataset. Chlorpyrifos exposure induced changes in metabolites linked to four major systems in the hepatocytes. These were energy metabolism, lipid metabolism, BCAA/glutathione metabolism and nucleotide metabolism.

**Table 3:** Biochemicals with interaction effects between chlorpyrifos (CPF) and vitamin E (Vit E).

							Two-Way ANOVA		
Super Pathway	Sub Pathway	Biochemical Name	Platform	Comp ID	KEGG	HMDB	CPF Main Effect P-value	Vit E Main Effect	CPF:Vit E Interaction P-value
Amino Acid	Phenylalanine and Tyrosine Metabolism	homogentisate	LC/MS neg	521	C00544	HMDB00130	0.0083		0.0370
	Methionine, Cysteine, SAM and Taurine Metabolism	homocysteine	GC/MS	40266	C00155	HMDB00742	0.8590		0.0143
Peptide	Dipeptide	alanylarginine	LC/MS neg	37096			0.0058		0.0291
		alanylisoleucine	LC/MS pos	37118			0.0038		0.0229
		alanylleucine	LC/MS pos	37093			0.0021		0.0347
		alanylphenylalanine	LC/MS pos	38679			0.0000		0.0334
		alanyltyrosine	LC/MS pos	37098			0.0000		0.0255
		alpha-glutamyltyrosine	LC/MS pos	40033			0.0004		0.0304
		leucylalanine	LC/MS pos	40010			0.0002		0.0462
		leucylarginine	LC/MS neg	40028			0.0000		0.0174
		methionylmethionine	LC/MS neg	40696			0.0031		0.0117
		seryltyrosine	LC/MS pos	42077			0.0031		0.0211
		valyllysine	LC/MS neg	41384			0.0000		0.0333
		valyltyrosine	LC/MS neg	40697			0.0002		0.0366
Lipid	Fatty Acid, Monohydroxy	13-HODE + 9-HODE	LC/MS neg	37752			0.6442		0.0093

**Fig 3:**
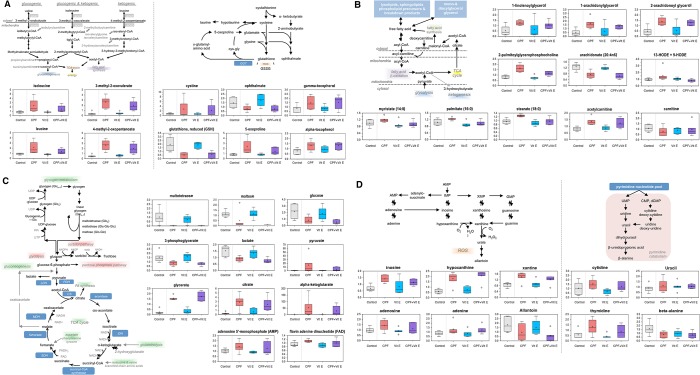
Predicted cellular outcome of chlorpyrifos exposure (100 μM) in Atlantic salmon hepatocytes based on affected metabolites. Effects on A) BCAA/glutathione metabolism B) lipid metabolism, C) energy metabolism and D) nucleotide metabolism. The figures show boxplots with median, upper and lower quartiles, max and min distribution, mean value (+) and extreme data points (ο) of scaled intensities. Values are normalized by Bradford protein concentration.

Chlorpyrifos exposure increased the accumulation of multiple free amino acids in the hepatocytes. Elevated levels of valine, isoleucine, and leucine in chlorpyrifos-treated cells were accompanied by an accumulation of the related alpha-keto acids 4-methyl-2-oxopentanoate and 3-methyl-2-oxovalerate (Fig. 3A). Vitamin E supplementation partially restored dipeptide levels in chlorpyrifos treated cells. Chlorpyrifos exposure depleted the levels of glutathione (GSH) and oxidized glutathione (GSSG), and increased the levels of cystine (formed by the oxidation of two cysteine molecules that covalently link via a disulfide bond) (Fig. 3A). In addition, increased levels of cystathionine, homocysteine and 5-oxoproline and diminished levels of 2-aminobutyrate and ophthalmate were seen in chlorpyrifos-exposed cells.

The long chain fatty acids palmitate and stearate accumulated in chlorpyrifos-treated hepatocytes compared to the controls (Fig. 3B). Vitamin E treatment was able to partially restore fatty acid levels to near control levels. The accumulation of these metabolites in chlorpyrifos-treated cells may reflect a change in the hydrolysis of complex lipids such as triglycerides as supported by the accumulation of monoacylglycerols such as 1-linoleoylglycerol and 1-arachidonylglycerol. The accumulation of these metabolites was modestly impacted by vitamin E supplementation. The polyunsaturated fatty acids docosapentaenoate, docosahexaenoate, and arachidonate were diminished in the presence of chlorpyrifos. Decreased levels of these metabolites indicate a conversion to lipid peroxidation products such as 13-HODE and 9-HODE that can be indicative of increased oxidative stress and are PPAR ligands associated with inflammatory hyperalgesia. Vitamin E supplementation also prevented chlorpyrifos-induced cholesterol accumulation in the cells.

For energy metabolism, chlorpyrifos exposed cells exhibited reduced levels of glycogen metabolites maltopentaose, maltotriose and maltose compared to controls (Fig. 3C). Vitamin E supplementation in the presence of chlorpyrifos was unable to restore glycogen metabolite levels. Alterations in glycogen may restrict glucose availability as evidenced by reduced levels of glucose in these cells. The glycolytic metabolites glucose 6-phosphate (G6P), fructose 1,6-bisphosphate, 3-phosphoglycerate, and lactate were reduced in the presence of the pesticide, regardless of vitamin E treatment. The TCA cycle intermediates citrate, alpha-ketoglutarate, and succinate significantly accumulated in the presence of chlorpyrifos.

Following chlorpyrifos exposure, the purine degradation products inosine, hypoxanthine, adenine, and xanthine accumulated in hepatocytes (Fig. 3D). Elevated levels of these metabolites can contribute to altered redox homeostasis since the generation of xanthine is accompanied by the production of hydrogen peroxide (H_2_O_2_). Purine catabolism may also be a component of the homeostatic response of the mitochondria to oxidative stress. Alternatively, these findings reflect increased nucleic acid availability potentially resulting from decreased cell growth and/or limited energy generation and excess AMP levels. Elevated levels of the pyrimidines cytidine and thymidine in chlorpyrifos treated cells were accompanied by an accumulation of uracil that may be indicative of an increase in nucleotide catabolism. These metabolic imbalances were not alleviated by vitamin E supplementation.

### Ingenuity pathway analysis (IPA)

**Fig 4:**
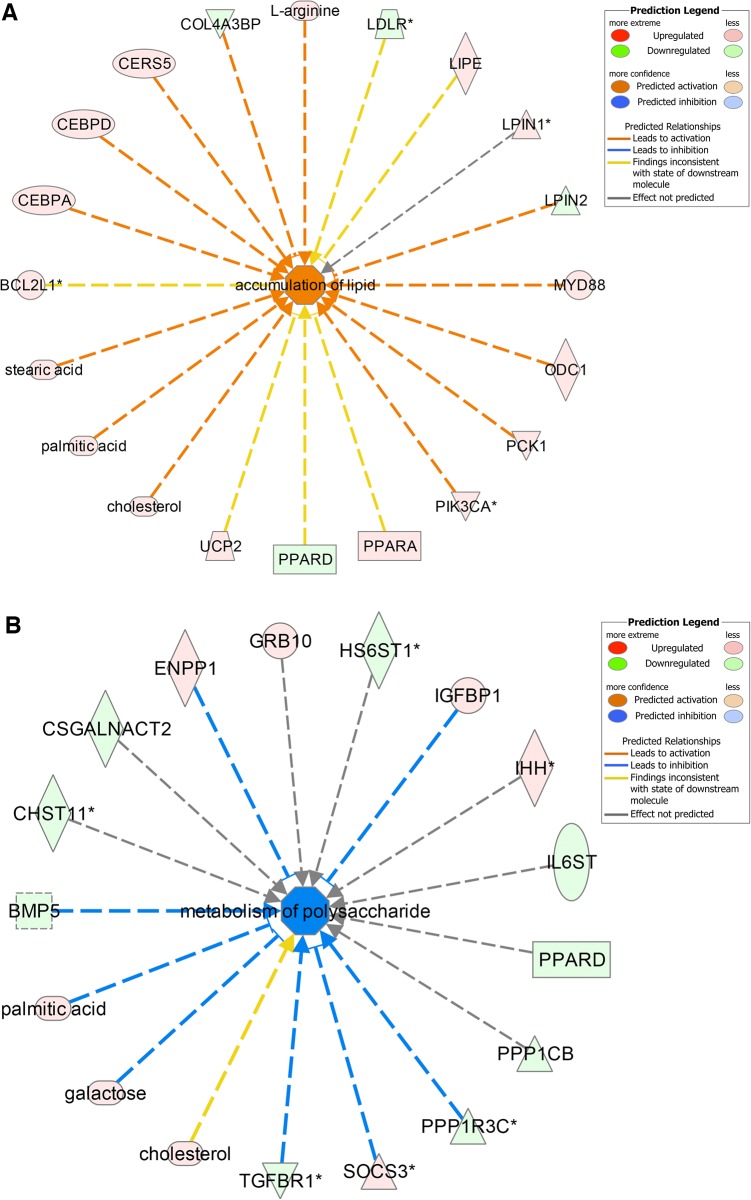
Ameliorating effects of vitamin E (100 μM) on chlorpyrifos (100 μM) toxicity in Atlantic salmon hepatocytes according to predicted activation and inhibition of biological functions. Based on combined lists of transcripts and metabolites. Data from IPA Core and Compare analyses. The legends show activation and inhibition states, and contribution of up-regulated and down-regulated molecules. A) Accumulation of lipid and B) Metabolism of polysaccharide.

IPA Core analysis and the IPA Compare function were used for evaluation of biological processes, pathways and networks. In order to use IPA, all identifiers must be recognized as mammalian homologs. Some fish-specific genes obviously cannot be given human ortholog names recognized by IPA, and thus cannot be included in IPA-Core analysis. To search for ameliorating effects of vitamin E on chlorpyrifos toxicity, the pairwise “control versus chlorpyrifos” and “control versus chlorpyrifos and vitamin E” comparisons were more closely examined. The number of identifiers included in the “control versus chlorpyrifos” and “control versus chlorpyrifos and vitamin E” IPA Core analyses were 640, 77 and 716, and 482, 64 and 565, respectively (transcripts, metabolites/biochemicals and combined). Heat maps generated with the IPA Compare function based on activation score from the transcriptomics analyses suggested that vitamin E supplementation ameliorated effects induced by chlorpyrifos related to cell survival and viability, bone cell formation-linked mechanisms, fatty acids (uptake of palmitic acid, concentration of acylglycerol) and amino acids (transport of L-amino acid), as well as numerous pathways linked to cancer and other mechanisms mainly of relevance for human health (S1 Table). A similar analysis by using the IPA Core analyses with metabolite identifiers suggested that vitamin E supplementation lowered the impact on free radical generation and detoxification (synthesis of nitric oxide, concentration of glutathione) and corresponding effects as suggested by the transcriptional data on fatty acid metabolism and amino acid transport. By combining the significantly affected transcripts and metabolites, in addition to effects on cell survival and viability, predicted activation of functions linked to fatty acids (accumulation of lipids (Fig. 4A), uptake of fatty acid, uptake of palmitic acid, accumulation of triacylglycerol) and predicted inhibition of functions linked to carbohydrates (metabolism of polysaccharide (Fig. 4B), transport and oxidation of monosaccharide, oxidation of D-glucose) were the most distinct effect of vitamin E supplementation.

## Discussion

This work shows that alpha tocopherol (vitamin E) to a modest degree can affect chlorpyrifos toxicity in Atlantic salmon liver cells. The cellular response as predicted by the metabolite outcome suggests that the main effects of chlorpyrifos exposure were on energy metabolism, lipid metabolism, BCAA/glutathione metabolism and nucleotide metabolism. Jak-STAT signaling was the most strongly affected pathway by chlorpyrifos exposure according to the transcriptional profiling. Vitamin E supplementation could only to a certain degree rescue the cells from the negative effects of the organophosphate pesticide.

In the nervous system, one of the main targets of chlorpyrifos toxicity in animals [10,31], the negative effects of the pesticide can lead to development of oxidative stress [32]. Numerous studies both with mammalian and fish models suggest that oxidative stress is one of the main outcomes of chlorpyrifos exposure [15]. As predicted, oxidative stress mechanisms were also affected by chlorpyrifos exposure in the Atlantic salmon hepatocytes. Depleted glutathione (GSH) and oxidized glutathione (GSSG) in the presence of chlorpyrifos indicate a decline in total glutathione availability, while elevated levels of cystine (formed by the oxidation of two cysteine molecules that covalently link via a disulfide bond) suggests free radical exposure. Alternatively, elevated cystathionine and homocysteine levels coupled with diminished 2-aminobutyrate and ophthalmate levels suggest a deficit in cysteine biogenesis and consequently the capacity to replenish glutathione. In support, higher levels of 5-oxoproline following pesticide treatment is indicative of the import and degradation of gamma-glutamyl amino acids to replenish GSH. Vitamin E administration was unable to restore cysteine metabolism in this study. Elevated accumulation of the purine degradation products inosine, hypoxanthine, adenine, and xanthine following chlorpyrifos exposure indicate altered redox homeostasis since the generation of xanthine is accompanied by the production of hydrogen peroxide (H_2_O_2_) [33]. Published studies also demonstrate that purine catabolism may be a component of the homeostatic response of the mitochondria to oxidative stress [34].

At the transcriptional level, chlorpyrifos exposure significantly affected the Gene Ontology “Response to oxidative stress” (GO:0006979) genes antioxidant 1 copper chaperone (ATOX1), protein phosphatase 1 regulatory subunit 15B (PPP1R15B) and nuclear factor erythroid 2-related factor 2 (NFE2L2). The latter gene, NFE2L2, is a transcription activator that binds to antioxidant response elements (ARE) in the promoter regions of target genes important for the coordinated regulation of genes in response to oxidative stress [35]. NFE2L2, which was downregulated by chlorpyrifos exposure, did not show altered transcription in cells co-treated with vitamin E. In addition, the B-cell translocation gene 1, anti-proliferative (BTG1) and NF-kappa-B 1 p105 subunit (NFKB1) genes were significantly affected by chlorpyrifos and vitamin A co-treatment but not by chlorpyrifos alone, while vitamin E supplementation counteracted the chlorpyrifos-induced ATOX1 response. The NFKB1 encodes a transcription factor present in almost all cell types involved in multiple cellular responses to stimuli such as cytokines and stress [35]. Its downregulation in cells co-treated with chlorpyrifos and vitamin E but not in pesticide-treated cells alone may indicate ameliorating effects of the antioxidant on mechanisms linked to apoptosis or proteasome-dependent degradation of proteins. Accordingly, the ATOX1 protein functions as an antioxidant against superoxide and hydrogen peroxide [35], and the differential transcription in cells given vitamin E may suggest a protective effect of the antioxidant. Chlorpyrifos exposure also induced pyruvate dehydrogenase kinase isozyme 2 (PDK2), a serine/threonine kinase functionally belonging in the Gene Ontology “Cellular response to reactive oxygen species” (GO:0034614) that plays a role in the regulation of cell proliferation and in resistance to apoptosis under oxidative stress [35]. No effect of vitamin E supplementation was however seen on PDK2 transcription in the cells. CuZn superoxide dismutase (SOD1) and catalase (CAT), suggested markers for longer-term chlorpyrifos exposure (15 days) in the Nile tilapia (*Oreochromis niloticus*) by Oruc [36], were not among the affected transcripts in cultured Atlantic salmon hepatocytes. To see effects on typical oxidative stress response genes, fish studies suggest that longer-term chlorpyrifos exposure is needed [37]. Overall, the transcriptional data including significantly affected pathways (S2 Dataset) suggest that the antioxidant had only a modest effect on chlorpyrifos-induced oxidative damage leading to apoptosis.

One of the most pronounced effects of chlorpyrifos exposure on metabolites was the accumulation of multiple free amino acids in the hepatocytes. The accumulation of multiple free amino acids in chlorpyrifos treated cells may reflect decreased utilization for protein synthesis or an increase in proteolysis considering multiple dipeptides significantly accumulated in these cells. Elevated levels of valine, isoleucine, and leucine in chlorpyrifos-treated cells were accompanied by an accumulation of the related alpha-keto acids 4-methyl-2-oxopentanoate and 3-methyl-2-oxovalerate that may also be indicative of degradation. These findings may reflect oxidative damage of proteins and subsequent degradation. Vitamin E supplementation partially restored dipeptide levels in chlorpyrifos treated cells and this was the strongest effect of vitamin E observed in the current study. Significant interaction effects between chlorpyrifos and vitamin E were observed for 12 dipeptides. Collectively, these findings are in agreement with published studies demonstrating chlorpyrifos induces oxidative stress in multiple model systems [36,37,38,39,40]. Alpha tocopherol supplementation at the studied concentration did not counteract these effects, suggesting that vitamin E, except its ability to partially restore dipeptide levels, has a relatively limited ability to rescue chlorpyrifos-induced oxidative stress in salmon hepatocytes.

Chlorpyrifos exposure affected the levels of several long chain fatty acids. The accumulation of these metabolites in chlorpyrifos treated cells indicates a change in the hydrolysis of complex lipids such as triglycerides as supported by the accumulation of monoacylglycerols such as 2-linoleoylglycerol and 1-arachidonylglycerol. Vitamin E treatment was able to partially restore the levels of these fatty acids to near control levels. Altered levels of lysolipids indicate a difference in membrane remodeling in the presence of pesticide. The accumulation of these metabolites was only modestly impacted by vitamin E supplementation suggesting a partial restoration of lipid homeostasis. Elevated lipid levels indicate increased eta-oxidation as suggested by the accumulation of acetylcarnitine (contributes to the movement of acetyl CoA in the mitochondria during lipid oxidation), in line with earlier mammalian studies [39]. In contrast to long chain fatty acids, the polyunsaturated fatty acids docosapentaenoate, docosahexaenoate, and arachidonate were diminished in the presence of chlorpyrifos. Lower polyunsaturated fatty acids indicate utilization of these precursors for the generation of pro- and anti-inflammatory eicosanoids. Furthermore, decreased levels of these metabolites may be indicative of conversion to lipid peroxidation products such as 13-HODE and 9-HODE that can be indicative of increased oxidative stress and are peroxisome proliferator-activated receptor (PPAR) ligands associated with inflammatory hyperalgesia. 13-HODE and 9-HODE are oxidative metabolites of the essential fatty acid linoleic acid [35] and these were the only lipid metabolism biochemicals that showed a significant interaction effect for chlorpyrifos and vitamin E. In a previous study in which Atlantic salmon hepatocytes were exposed to chlorpyrifos [22], several putatively annotated eicosanoids in different lipid metabolism pathways were affected by the exposure. We were however not able to reproduce the effect on these metabolites in the current experiment, although the suggested overall effect on lipid metabolism was the same. A predicted inhibition of release of eicosanoids, due to altered levels of arachidonic acid, D-glucose, D-mannose, glycine and cholesterol after chlorpyrifos exposure, was enhanced by vitamin E supplementation in the current study. Compared to the cells exposed to chlorpyrifos alone, vitamin E supplementation contributed to a predicted inhibition of eicosanoid synthesis.

It is well known that many lipid-soluble contaminants, including organophosphorous pesticides, can contribute to accumulation of lipids in fish liver [41]. In crucian carp (*Carassius auratus gibelio*), for example, Xu et al. [42] found increased levels of triglycerides in the liver after exposure to the organophosphorous pesticide trichlorfon. Recent studies have shown a strong correlation between chemical exposure and steatosis (fatty liver) [43]. We have earlier shown that the organochlorine insecticide endosulfan induces steatosis in Atlantic salmon hepatocytes [44], while Sun et al. [45] showed a similar response in male rockfish (*Sebastiscus marmoratus*) exposed to the triazole-containing fungicide paclobutrazol. As shown in Fig. 3A, a number of genes who’s proteins are linked to increased accumulation of lipids displayed differential expression in this study. Of these, it is interesting to note that while vitamin E prevented the chlorpyrifos-induced increased expression of PPARA, no effect was seen on the PPAR beta 1A (PPARB1A, an ortholog to mammalian PPARD).

Impaired metabolism of a number of drugs has been linked to steatosis, suggesting an association between increased lipid deposition and impaired cytochrome P450 (CYP) enzymes [46]. In carp (*Cyprinus carpio*), it has been shown that long-term exposure to chlorpyrifos results in CYP1A mRNA induction and increased EROD activity in the liver [47]. Surprisingly, the highly inducible CYP1A gene was not among the gene transcripts significantly induced by chlorpyrifos exposure in the exposed liver cells. According to the transcriptional data, only two gene transcripts, both annotated to cytochrome P450 2K1, were differentially affected by chlorpyrifos exposure. Co-treatment with vitamin E prevented this effect.

One possible explanation on how vitamin E might help prevent accumulation of lipids in fish liver is disturbance of cholesterol transport and uptake, as no cholesterol accumulation was observed in pesticide-treated cells. The transcriptional data showed that chlorpyrifos-induced downregulation of low-density lipoprotein receptor (LDLR) was prevented by vitamin E treatment. LDLR binds low-density lipoprotein (LDR), the major cholesterol-carrying lipoprotein of plasma, and transports it into cells by endocytosis [35]. Similarly, hormone-sensitive lipase (LIPE), which primarily hydrolyzes stored triglycerides to free fatty acids [35], and fatty acid-binding protein 3 (FABP3) which is involved in uptake, intracellular metabolism and transport of long-chain fatty acids [35], were upregulated by chlorpyrifos treatment but not in cells co-treated with vitamin E. The antioxidant appears to have less effect on membrane-associated gene proteins such as sphingosine 1-phosphate receptor 2 (S1PR2) and phospholipase A2, Group XIIB (PLA2G12B) that were affected by chlorpyrifos treatment.

One of the main findings in the current study was that chlorpyrifos exposure affected energy metabolism in the cells. Chlorpyrifos exposed cells exhibited reduced levels of the several glycogen metabolites. Vitamin E supplementation in the presence of chlorpyrifos was unable to restore glycogen metabolite levels. Alterations in glycogen may restrict glucose availability as evidenced by reduced levels of glucose in the cells. Consequently, the glycolytic metabolites glucose 6-phosphate (G6P), fructose 1,6-bisphosphate, 3-phosphoglycerate, and lactate were reduced in the presence of the pesticide. Alternatively, lower glucose levels may suggest a difference in transport as supported by rat studies showing that chlorpyrifos inhibits glucose uptake in liver [38]. Consequently, the glycolytic metabolites glucose 6-phosphate (G6P), fructose 1,6-bisphosphate, 3-phosphoglycerate, and lactate were reduced in the presence of the pesticide, regardless of vitamin E treatment. Chronic exposure to organophosphate pesticides can lead to deleterious effects on carbohydrate metabolism. Carbohydrate-metabolizing organs, such as the liver, can be affected by organophosphate pesticides through altered glycolysis, gluconeogenesis, glycogenesis, and glyconeogenesis [48]. Fig. 3B summarizes which gene transcripts and metabolites that were involved in polysaccharide metabolism as identified by the IPA analyses. In contrast to glycolytic intermediates, the TCA cycle intermediates citrate, alpha-ketoglutarate, and succinate significantly accumulated in the presence of chlorpyrifos regardless of vitamin E supplementation. This imbalance in TCA metabolites may be indicative of mitochondrial dysfunction and potentially reflect oxidative inhibition of mitochondrial enzymes such as aconitase. Similarly, the reduced activity of liver mitochondrial isocitrate dehydrogenase (IDH) has been correlated with organophosphate toxicity in other models [49,50]. Taken together, these findings suggest that chlorpyrifos may limit the energetic capacity of salmon hepatocytes, and that vitamin E supplementation has limited ability to restore these mechanisms.

Interestingly, the IPA evaluation suggested that several pathways normally linked to the central nervous system were affected in liver by chlorpyrifos exposure. This finding is not surprising given that one of the main effects of chlorpyrifos exposure in animals is inhibition of AChE activity [31]. Two major cholinesterase enzymes are found in fish. AChE is most common in brain and muscle, while butyrylcholinesterase (BChE) is found mainly in plasma and in the liver [51]. Relatively high AChE activity has been reported in fish liver [52]. A significant reduction in hepatic AChE activity and mRNA level has been reported in fish exposed to chlorpyrifos [53]. The significant effect seen on Jak-STAT signaling by chlorpyrifos exposure may be due to damage inflicted by the bioactivated oxon on liver cholinesterases or reflect alterations in protein phosphorylation pathways [54]. A similar response has been reported in brain of mice exposed to chlorpyrifos [55].

Chlorpyrifos is a known endocrine disruptor. Two transcripts annotated to genes associated with the Gene Ontology “Response to estrogen stimulus” (GO:0043627) were significantly affected by pesticide treatment. Both the TGF beta receptor-1 (TGFBR1) and the Cbp/p300-interacting transactivator 2 (CITED2) were downregulated by chlorpyrifos exposure. Vitamin E supplementation did however not alter this response. In addition, two gene transcripts associated with the Gene Ontology “Estrogen receptor binding” (GO:0030331) were differentially regulated. ATP-dependent RNA helicase DDX5 (DDX5) was upregulated by chlorpyrifos exposure, while nuclear receptor-interacting protein 1 (NRIP1) was downregulated by the pesticide. The latter response was ameliorated by vitamin E treatment, however, the TGF-beta signaling pathway response was not modified by the antioxidant. These findings suggest that chlorpyrifos may affect mechanisms linked to endocrine disruption in Atlantic salmon liver cells.

The transcriptional and metabolite profiling approaches give complementary outcomes, supported by the IPA analyses. According to the transcriptional data, Jak-STAT signaling, Protein processing in endoplasmic reticulum and Pathways in cancer were the three most significantly affected KEGG pathways by chlorpyrifos exposure. These results suggest that the pesticide induced cellular damage leading to apoptosis. Significant effects were seen on several DEGs belonging to the Gene Ontology “Induction of apoptosis” (GO:0006917). These included genes such as GTP cyclohydrolase 1 (GCH1), serine/threonine-protein kinase 24 (STK24), Krueppel-like factor 10 (KLF10), rho GTPase-activating protein 7 (DLC1), TGFBR1, etoposide-induced protein 2,4 homolog (EI24), mitochondrial ubiquitin ligase activator of NF-kB (MUL1), as well as apoptosis regulator BCLX. This result fits nicely with the observed accumulation of free amino acid as shown by the metabolomics data. Branched amino acids, such as valine, isoleucine, and leucine discussed above, can induce apoptosis in animal cells [56]. Jak-STAT signaling has also been linked to altered lipid metabolism [57]. As described above, one of the main effects of chlorpyrifos exposure was induction of steatosis. Intracellular fat accumulation has been shown to induce endoplasmatic stress and disrupt Jak-STAT signaling in human cell cultures [58], further demonstrating the complementary nature of the two methods. As a key regulator of numerous cytokines, growth factors and hormones, the JAK-STAT pathway [57] can have a profound effect on energy metabolism in vivo [57]. This study shows a similar outcome in cell cultures. Applied together, transcriptional and metabolite profiling therefore appear to be complementary by nature.

In conclusion, this study shows that vitamin E supplementation may help protect cells against chlorpyrifos-induced toxicity by restoring dipeptide levels, preventing accumulation of lipids and modifying carbohydrate metabolism. Both the transcriptional and metabolite profiling suggest that this effect is relatively modest, and that vitamin E supplementation will not rescue the cells completely. Although chlorpyrifos exposure clearly induced oxidative stress in the hepatocytes, the protective effect of vitamin E was minor. The study also demonstrates the complementary nature of transcriptional and metabolite profiling, with the more detailed transcriptional responses supplementing the cellular outcome as predicted by the metabolite changes.

## Supporting Information

S1 Table Heat map generated with the IPA Compare function using both the transcriptomics and metabolomics data. (XLSX)

S1 Dataset Pairwise comparisons of transcriptional data. (XLSX)

S2 Dataset Statistically significant KEGG pathways from gene set enrichment analysis. (XLSX)

S3 Dataset Pathway-specific heat maps of affected metabolites. (XLSX)
